# Valor Prognóstico da Taquicardia Ventricular não Sustentada na Miocardiopatia Hipertrófica em Coorte Brasileira: Comparação com Literatura Mundial

**DOI:** 10.36660/abc.20240399

**Published:** 2025-05-13

**Authors:** Diego Araújo Silva, Edmundo Arteaga-Fernandez, Viviane Tiemi Hotta, Charles Mady, Barbara Ianni, Felix Ramires, Luciano Nastari, Fábio Fernandes, Juliano Novaes Cardoso

**Affiliations:** 1 Faculdade Santa Marcelina Curso de Medicina São Paulo SP Brasil Faculdade Santa Marcelina Curso de Medicina, São Paulo, SP – Brasil; 2 Hospital das Clínicas Faculdade de Medicina Universidade de São Paulo São Paulo SP Brasil Instituto do Coração do Hospital das Clínicas da Faculdade de Medicina da Universidade de São Paulo, São Paulo, SP – Brasil; 3 Faculdade de Medicina Universidade de São Paulo São Paulo SP Brasil Faculdade de Medicina da Universidade de São Paulo, São Paulo, SP – Brasil; 4 Hospital Santa Marcelina São Paulo SP Brasil Hospital Santa Marcelina, São Paulo, SP – Brasil

**Keywords:** Cardiomiopatia Hipertrófica, Fatores de Risco, Taquicardia Ventricular

## Abstract

**Fundamento:**

Na miocardiopatia hipertrófica (MCH), é conhecida a associação entre a taquicardia ventricular não sustentada (TVNS) e o risco de morte súbita.

**Objetivos:**

Avaliar a incidência de TVNS pelo Holter 24 h em pacientes com MCH em coorte brasileira e correlacionar com suas características e evolução.

**Métodos:**

No presente estudo retrospectivo de pacientes com MCH, avaliamos pelo Holter 24 h a presença de TVNS longas e rápidas (≥ 10 batimentos e frequência ≥ 130 bpm) ou a presença de pelo menos 3 episódios de TVNS com ≥ 3 batimentos e frequência ≥ 120 bpm. As variáveis contínuas foram apresentadas em médias aritméticas e desvio padrão e as variáveis categóricas em frequências absolutas e relativas. Foi considerado significativo p < 0,05.

**Resultados:**

Incluímos 763 pacientes, 53,5% do sexo masculino. A idade média foi de 52,6 anos ± 16,7. A presença de TVNS foi evidenciada em 10% (76 pacientes). Destes, apenas 11 (1,4%) apresentaram TVNS ≥ 10 batimentos e frequência ≥ 130 bpm. Não houve diferença na relação da TVNS com sexo, septo > 30 mm, idade ≥ 40 anos, dose de betabloqueador e presença de fibrilação atrial. No grupo com TVNS a mortalidade por todas as causas ao longo de 15 anos foi observada em 26,3% versus 15,9% no grupo sem TVNS (p = 0,021).

**Conclusões:**

A presença de TVNS no Holter 24 h ocorreu em 10 % dos pacientes. As TVNS longas e rápidas foram raras. A presença de TVNS foi associada à maior mortalidade geral ao longo do seguimento.

## Graphical abstract

**Figura Central:**
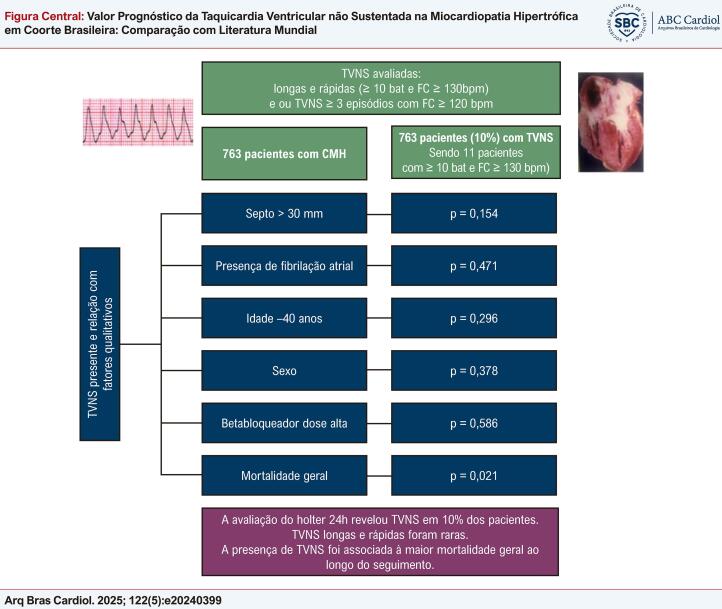
Valor Prognóstico da Taquicardia Ventricular não Sustentada na Miocardiopatia Hipertrófica em Coorte Brasileira: Comparação com Literatura Mundial Valor prognóstico da taquicardia ventricular não sustentada na miocardiopatia hipertrófica em coorte brasileira. FC: frequência cardíaca; MCH: miocardiopatia hipertrófica; TVNS: taquicardia ventricular não sustentada.

## Introdução

A miocardiopatia hipertrófica (MCH) é uma doença que cursa com hipertrofia do ventrículo esquerdo, com expressão morfológica restrita ao coração. Sua presença envolve grande variabilidade de fenótipos clínicos, visto que grande parte dos pacientes são frequentemente assintomáticos ou oligossintomáticos. No entanto, a doença pode cursar com morte súbita, isquemia miocárdica, limitação funcional, disfunção sistólica do ventrículo esquerdo, fibrilação atrial com risco aumentado de eventos trombóticos e arritmias ventriculares.^1-8^ É a causa mais comum de morte súbita em indivíduos jovens e em atletas, e a maioria dos episódios está relacionado à arritmia ventricular. Dentre os fatores de risco para morte súbita, temos a presença de taquicardia ventricular não sustentada (TVNS), idade mais jovem, história familiar de morte súbita precoce, síncope, espessura septal, gradiente via saída do ventrículo esquerdo, tamanho átrio esquerdo e quantidade de fibrose. A TVNS foi associada em alguns estudos com o maior grau de comprometimento do ventrículo esquerdo, maior índice de massa, além de ser mais frequente em casos que apresentam maior espessura parietal.^2,5,7-15^

Recomenda-se a monitorização com Holter na avaliação inicial e como parte do acompanhamento periódico a cada 1 ou 2 anos. Isso ajuda na avaliação do risco de morte súbita cardíaca e na orientação do manejo de arritmias. Alguns critérios podem tornar a TVNS mais relevante, dentre eles as que são mais frequentes, as mais rápidas ou ainda as mais longas.^4-8^ A incidência de TVNS tem sido relatada em 20% a 54% de pacientes.^5-10,13-20^

Devido à importância e aos riscos das arritmias na MCH, o presente estudo tem como objetivo avaliar a incidência de TVNS ao Holter de 24h em pacientes com MCH em coorte brasileira, comparando com dados da literatura mundial, e correlacionar com suas características e mortalidade por todas as causas.

## Métodos

O estudo avaliou uma coorte retrospectiva de pacientes com o diagnóstico de MCH no ambulatório de um hospital terciário. O diagnóstico ecocardiográfico foi definido pela espessura miocárdica diastólica ≥ 15 mm em qualquer localização do ventrículo esquerdo, ou ≥ 13 mm em indivíduos com história familiar de parentes de primeiro grau com diagnóstico confirmado de MCH. Foram considerados os seguintes critérios de exclusão: pacientes menores de 16 anos, com estenose aórtica, presença de outras causas que justificassem a hipertrofia do miocárdio e que não possuíam Holter da instituição, ou que não apresentavam o exame do Holter completo disponível no prontuário eletrônico para análise. A definição do tamanho da amostra foi realizada por conveniência.

Foram analisados dados demográficos, clínicos e laboratoriais, além do eletrocardiograma de repouso, do ecocardiograma e do Holter 24 h. No ecocardiograma, as medidas das cavidades e dimensões cardíacas foram realizadas pelo modo bidimensional no corte paraesternal longitudinal. O seguimento clínico foi realizado pelos médicos assistentes de acordo com a rotina do serviço.

Na avaliação do Holter 24 h, consideramos as TVNS longas e rápidas (≥ 10 batimentos com frequência ≥ 130 bpm) ou a presença de ≥ 3 episódios de TVNS, com no mínimo 3 batimentos e frequência ≥ 120 bpm. Foi avaliado o uso de betabloqueador e a dose utilizada. Consideramos dose alta a utilização de atenolol ≥ 50 mg/dia; propranolol ≥ 40 mg/dia; metoprolol ≥ 50mg/dia; carvedilol ≥ 12,5 mg/dia.

### Análise estatística

Para processamento estatístico dos dados foram utilizados os programas SPSS V26 (2019), Minitab 21.2 (2022) e Office Excel 2010. As variáveis contínuas foram apresentadas em médias aritméticas e desvio padrão e as variáveis categóricas em frequências absolutas e relativas. Utilizamos o teste t de Student não pareado para as variáveis contínuas e o teste qui-quadrado para as variáveis categóricas. O teste de Shapiro-Wilk foi utilizado para testar a normalidade das variáveis contínuas. As curvas de sobrevida foram analisadas com o método de Kaplan-Meier, utilizando o teste de log-rank. O nível de significância adotado na análise estatística foi de 5%. O estudo foi aprovado pelo comitê de ética da instituição.

## Resultados

Foram analisados em banco de dados 2572 pacientes com hipertrofia miocárdica, sendo confirmada a MCH em 1663, dos quais 1593 apresentavam idade igual ou superior a 16 anos. Desse grupo, 763 apresentavam o exame de Holter disponível no prontuário na sua forma completa e realizado pela instituição, possibilitando assim, uma análise mais apurada dos dados. No grupo avaliado, 63 pacientes (8,3%) eram portadores de marcapasso e 52 pacientes (6,8%) eram portadores de cardioversor-desfibrilador implantável (CDI). As características dos pacientes incluídos estão descritas na Tabela 1.

**Tabela 1:** – Características clínicas (763 pacientes)

Variável	763 pacientes % ou média (DP)
Sexo masculino (%)	408 (53,5%)
Idade, média (DP)	52,6 (16,7)
PAS mmHg, média (DP)	113,2 (34,7)
PAD mmHg, média (DP)	70,5 (21,3)
FC (bpm), média (DP)	64,8 (20,3)
FEVE, média (DP)	66% (7,2)
Fibrilação atrial (%)	46 (6,0%)
Glicose, média (DP)	107,5 (31,1)
Hemoglobina, média (DP)	14,1 (2,0)
Ureia, média (DP)	41,5 (22,8)
Creatinina, média (DP)	1,1 (0,6)
Potássio, média (DP)	4,4 (0,5)
LDL, média (DP)	108,3 (35,7)
HDL, média (DP)	47,0 (14,0)
BNP, média (DP)	464,9 (767,2)

Os valores são expressos por média ± DP ou frequência e porcentagem. DP: desvio padrão; FC: frequência cardíaca; FEVE: fração de ejeção do ventrículo esquerdo; PAD: pressão arterial diastólica; PAS: pressão arterial sistólica.

O Holter 24 h (Tabela 2) revelou frequência cardíaca média de 70,3 ± 11 bpm. Dentre os pacientes avaliados, 50,5% apresentaram frequência cardíaca média no Holter < 70 bpm (385 pacientes) e 13,4% < 60 bpm (102 pacientes). A presença de TVNS foi evidenciada em 76 pacientes (10%). Em apenas 11 pacientes (1,4%) o Holter revelou TVNS com 10 ou mais batimentos e frequência ≥ 130 bpm. Somente 4 pacientes (0,5%) apresentaram TVNS com frequência cardíaca ≥ 200 bpm; entretanto, foram episódios curtos, com no máximo 4 batimentos.

**Tabela 2:** – Resultados do Holter 24 h (n = 763)

Parâmetros	Média (DP)
FC mínima (bpm), média (DP)	49,3 (7,7)
FC média (bpm), média (DP)	70,3 (11,0)
FC máxima (bpm), média (DP)	117,9 (24,6)
ESV (total), média (DP)	761,4 (3853,5)
Número de batimentos em 24 horas, média (DP)	94222,4 (16590,6)
TV sustentada (%)	1 (0,1%)
TVNS (%)	76 (10,0%)

ESV: extrassístoles ventriculares; FC: frequência cardíaca; TV: taquicardia ventricular; TVNS: taquicardia ventricular não sustentada.

O ecocardiograma revelou que a fração de ejeção do ventrículo esquerdo (FEVE) média foi de 66% ± 7,2%. A forma obstrutiva para via de saída do ventrículo esquerdo foi evidenciada em 219 pacientes (28,7%), enquanto 43 (5,6%) apresentaram espessura septal acima de 30 mm. A avaliação da prescrição completa foi possível em 439 pacientes, sendo que 351 pacientes (80%) utilizaram o betabloqueador. Destes que utilizaram betabloqueador, 212 pacientes (60,4%) utilizaram doses altas. As outras medicações utilizadas foram verapamil em 42 pacientes (9,6%), amiodarona em 95 pacientes (21,6%) e inibidor da enzima conversora da angiotensina ou bloqueador do receptor da angiotensina em 244 pacientes (55,6%).

**Figura 1:**
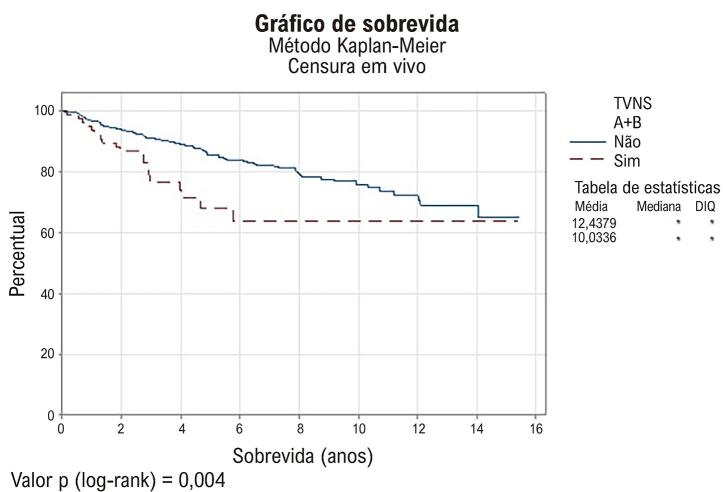
– Curva de sobrevida para TVNS. DIQ: desvio interquartil; TVNS: taquicardia ventricular não sustentada.

Não houve correlação entre a presença de TVNS com sexo, tamanho de septo > 30 mm, idade ≥ 40 anos, dose alta ou não de betabloqueador e a presença de fibrilação atrial (Tabela 3). Na Figura Central demonstramos um resumo com a incidência de TVNS em nossa coorte e a relação com os fatores qualitativos. No grupo com TVNS, a mortalidade por todas as causas ao longo de 15 anos foi observada em 26,3% (20 pacientes) versus 15,9% (109 pacientes) no grupo sem TVNS (p = 0,021). Na Figura 1 demonstramos a curva de sobrevida pelo método de Kaplan-Meier.

**Tabela 3:** – Relação entre TVNS e fatores qualitativos

		Com TVNS		Sem TVNS		Total		Valor p
		N	%	N	%	N	%	
Septo acima de 30 mm	Não	69	90,8%	651	94,8%	720	94,4%	0,154
	Sim	7	9,2%	36	5,2%	43	5,6%	
BB dose alta	Não	25	55,6%	202	51,3%	227	51,7%	0,586
	Sim	20	44,4%	192	48,7%	212	48,3%	
FA	Não	70	92,1%	647	94,2%	717	94,0%	0,471
	Sim	6	7,9%	40	5,8%	46	6,0%	
Idade	< 40 anos	15	19,7%	173	25,2%	188	24,6%	0,296
	≥ 40 anos	61	80,3%	514	74,8%	575	75,4%	
Óbito	Não	56	73,7%	578	84,1%	634	83,1%	0,021
	Sim	20	26,3%	109	15,9%	129	16,9%	
Sexo	Feminino	39	51,3%	316	46,0%	355	46,5%	0,378
	Masculino	37	48,7%	371	54,0%	408	53,5%	

BB: betabloqueador; FA: fibrilação atrial; TVNS: taquicardia ventricular não sustentada**.**

A avaliação do ritmo cardíaco, pelo eletrocardiograma de repouso e pelo Holter 24 h revelou ritmo de fibrilação atrial em 46 pacientes (6,0%), sendo que os pacientes com átrio esquerdo com tamanho > 40 mm tiveram prevalência maior de fibrilação atrial (64,8% dos pacientes em ritmo sinusal versus 91,1% dos pacientes em ritmo de fibrilação atrial com tamanho de átrio esquerdo > 40 mm, p < 0,001).

## Discussão

Este estudo revelou a presença de TVNS em 10% dos casos (76 pacientes), sendo a mortalidade geral, ao longo de 15 anos, maior no grupo com TVNS (26,3% versus 15,9%, p = 0,021). Não houve diferença estatisticamente significativa na relação da TVNS com sexo, tamanho de septo > 30 mm, idade ≥ 40 anos, dose alta ou não de betabloqueador e presença de fibrilação atrial. A presença de TVNS longas e rápidas teve uma incidência muito baixa, sendo evidenciada em apenas 1,4% dos pacientes (11 pacientes), conforme demonstramos na Figura Central.

Na avaliação do paciente com MCH, a presença de taquicardia ventricular está associada à morte súbita. O Holter é o exame recomendado para avaliar a presença de arritmias e ajudar na estratificação do paciente. A presença de taquicardia ventricular sustentada já é indicativa para o implante de CDI.^1,2,7^ Nosso estudo revelou que a incidência de taquicardia ventricular sustentada observada no Holter 24 h foi muito rara, sendo identificada em apenas 1 paciente. Provavelmente, por ser um exame com duração de apenas 24 horas, nem sempre conseguimos identificar este tipo de arritmia. Já a presença de TVNS é um achado um pouco mais frequente conforme dados já publicados.^6-10^

A TVNS faz parte dos fatores de risco maiores para morte súbita cardíaca, ao lado da história familiar de morte súbita, síncope inexplicada, espessura septal acima de 30 mm, FEVE < 50%, aneurisma apical e fibrose acima de 15%. Estudos revelam que a incidência de TVNS pode variar e tem sido relatada em 20% a 54% dos pacientes com MCH. Nosso estudo revelou uma incidência menor dessa arritmia, com 10% apresentando TVNS. Uma das hipóteses para essa diferença está na caracterização do tipo de TVNS significativa. Devemos dar maior peso às arritmias mais longas, frequentes e rápidas, critérios que utilizamos em nosso estudo. Entretanto, alguns estudos incluem as TVNS menos significativas.^6-10,13,20^

Em um estudo sobre a TVNS na MCH, Monserrat et al.^6^ avaliaram 531 pacientes. O Holter 24 h revelou 19,6% com TVNS. A TNVS foi relacionada com aumento da morte súbita em pacientes com menos de 30 anos. A razão de chances de morte súbita em pacientes com ≤ 30 anos de idade com TVNS foi de 4,35 (intervalo de confiança 95%: 1,54 a 12,28; p = 0,006) em comparação com 2,16 (intervalo de confiança 95%: 0,82 a 5,69; p = 0,1) em pacientes com mais de 30 anos. Em nosso estudo a incidência de TVNS foi bem menor; entretanto, isto está relacionado com a caracterização da TVNS. Nós utilizamos apenas as TVNS mais significativas, enquanto o estudo de Monserrat et al.^6^ considerou qualquer tipo de TVNS. Essa diferença nos critérios utilizados para definirmos TVNS significativa pode ser um dos motivos para a diferença na incidência que encontramos.

Wang et al.^9^ avaliaram 160 pacientes que implantaram CDI, sendo que 94 realizaram Holter de 24 a 48 horas antes do implante. O resultado desse estudo revelou que 54% do total (86 pacientes) apresentaram TVNS, seja pela avaliação do Holter pré-implante ou pela monitorização do CDI após o implante. Um fato que deve ser levado em consideração é que no trabalho de Wang et al.,^9^ o tempo de monitorização foi maior, incluindo dados da monitorização com dispositivo invasivo. Além disso, eles selecionaram apenas pacientes de maior risco para arritmia. Nosso estudo é de uma população geral com MCH e utilizamos o Holter 24 h, o que pode justificar a diferença na incidência de TVNS. Esse é um outro motivo que justifica a menor incidência de TVNS que encontramos em nosso estudo. Avaliar a incidência de TVNS em pacientes de alto risco para arritmia e que já necessitam de CDI aumenta muito a chance de encontramos tal arritmia. Além disso, realizar uma monitorização contínua com dispositivo implantável também irá aumentar a chance em detectar arritmias, pois o tempo de monitorização é muito maior, comparado com o de um Holter 24 h.

Na diretriz europeia,^7^ a TVNS é pontuada no escore de risco para morte súbita, quando apresenta ≥ 3 batimentos ventriculares consecutivos e ≥ 120 bpm com duração < 30 s. Na diretriz brasileira de dispositivos,^5^ a TVNS é pontuada quando ocorre a presença de 3 ou mais episódios, com no mínimo 3 batimentos ventriculares repetitivos ou pelo menos 1 episódio com ≥ 10 batimentos e frequência ≥ 130 bpm. Já na diretriz americana,^2^ a TVNS entra como um critério independente, mas com grau de recomendação IIb e sugere dar maior peso às TVNS mais frequentes (≥ 3), longas (≥ 10 batimentos) ou rápidas (≥ 200 bpm). A diretriz brasileira^1^ sugere valorizar os episódios de TVNS frequentes (≥ 3), mais longos (≥ 10 batimentos) e mais rápidos (≥ 200 batimentos por minuto). Portanto, é importante identificar a presença das TVNS mais significativas e correlacionar com o risco. Em nosso estudo, apenas 11 pacientes (1,4%) apresentaram TVNS com 10 ou mais batimentos e frequência ≥ 130 bpm. Quando avaliamos as taquicardias com frequência muito alta, encontramos somente 4 pacientes (0,52%) com frequência ≥ 200 bpm, entretanto, com no máximo 4 batimentos. Na diretriz americana,^4^ um dos critérios de risco para morte súbita é a presença de TVNS com frequência ≥ 200 bpm. Como podemos observar em nossa coorte, a incidência de TVNS tão rápidas foi baixa. Portanto, é um critério que em nossa clínica diária iremos observar muito pouco.

Episódios de taquicardia ventricular sustentada estão mais claramente associados à morte súbita; entretanto, os dados são menos robustos em demonstrar que a presença isolada de TVNS seja um fator de risco independente. Por outro lado, o risco aumenta na presença de modificadores de risco, especialmente fibrose do ventrículo esquerdo.^8-10,21^ Em nosso estudo, a presença de TVNS foi um marcador de pior prognóstico na avaliação da mortalidade geral. Entretanto, não avaliamos a presença de fibrose e também não avaliamos especificamente a mortalidade cardiovascular. Em estudo realizado por Monserrat et al.,^6^ além da mortalidade cardiovascular, eles também avaliaram a mortalidade por todas as causas. Esse estudo revelou que nos pacientes com menos de 30 anos, a sobrevida foi menor no grupo TVNS (sobrevida cumulativa em 5 anos 61,3% [intervalo de confiança 95%: 41,1 a 81,5] com TVNS versus 93,5% [intervalo de confiança 95%: 89,4 a 97,6] sem TVNS; p = 0,0001). Eles concluíram que a TVNS foi associada a um aumento substancial na mortalidade por todas as causas e no risco de morte súbita em pacientes jovens com MCH. Este risco foi independente da frequência, duração e frequência cardíaca dos episódios de TVNS. Nosso estudo contemplou apenas a mortalidade geral, não mostrou diferença com a idade e revelou mortalidade geral maior nos pacientes com TVNS. Esses achados ajudam a sedimentar que a detecção de TVNS significativas em pacientes com MCH auxilia na estratificação do risco de eventos arrítmicos graves, embora a decisão sobre o implante de um CDI deva considerar múltiplos fatores.

Os betabloqueadores são considerados como terapia de primeira linha para controle de sintomas, tais como dispneia e dor torácica. Os bloqueadores de canal de cálcio não dihidropiridínicos, como verapamil e diltiazem, são alternativas de segunda linha para pacientes que não toleram ou têm contraindicação ao uso de betabloqueadores.^1,2,7^ Entretanto, não há dados randomizados e controlados para apoiar o uso de medicamentos antiarrítmicos para a prevenção de morte súbita na MCH. Em nosso estudo o uso do betabloqueador foi frequente, com 80% dos pacientes utilizando a medicação, sendo que 60,4% dos pacientes utilizaram doses altas. Bloqueador do canal de cálcio (verapamil) foi utilizado em 9,6% dos pacientes (42 pacientes). Em nosso estudo, a dose de betabloqueador não teve relação com a presença de TVNS.

A FEVE na MCH é usualmente normal ou aumentada e a espessura septal acima de 30 mm pode estar relacionada com a gravidade da doença.^2,5,11,21-24^ Nossa população apresentou uma FEVE média de 66% ± 7,2%. Além disso, apenas 5,6% dos pacientes apresentaram espessura septal acima de 30 mm, e estes pacientes não apresentaram maior número de TVNS.

Nosso estudo revelou uma incidência de TVNS mais baixa quando comparado aos dados da literatura.^5-11,13,20-23,25^ Isto pode estar relacionado ao tipo de população estudada e também aos critérios de inclusão para considerar uma TVNS significativa. Novos estudos prospectivos devem ser realizados para correlacionar a evolução cardiovascular e a presença de arritmia ventricular complexa em conjunto com outros fatores de risco.

### Limitações

Este foi um estudo unicêntrico, retrospectivo e realizado em hospital terciário. Em se tratando de amostragem de conveniência e sem estimativa de tamanho amostral, qualquer inferência estatística é exploratória. Por se tratar de um estudo retrospectivo, fomos limitados pelas informações avaliadas. Não avaliamos as comorbidades. Em muitos pacientes não tivemos acesso à prescrição completa, por isso a avaliação dos medicamentos utilizados foi em apenas uma parte dos pacientes incluídos. Outra limitação é que não tivemos acesso à causa da morte de cada paciente e por isso avaliamos a mortalidade por todas as causas.

## Conclusão

O presente estudo revelou a presença de TVNS em 10% dos casos, sendo a mortalidade geral ao longo de 15 anos maior no grupo com esta arritmia. Não houve diferença estatisticamente significativa na relação da TVNS com o sexo, tamanho de septo > 30mm, idade ≥ 40 anos, dose de betabloqueador e presença de fibrilação atrial. A presença de TVNS longas e rápidas teve uma incidência muito baixa (1,4% dos casos).
