# Supplementary material for: Valor Prognóstico da Taquicardia Ventricular não Sustentada na Miocardiopatia Hipertrófica em Coorte Brasileira: Comparação com Literatura Mundial
Source: Arq Bras Cardiol. 2025 May 13;122(5):e20240399. [Article in Portuguese] doi: 10.36660/abc.20240399 (PMC12129476; doi:10.36660/abc.20240399)
Supplement: Translation

# Translation: Prognostic Value of Nonsustained Ventricular Tachycardia in Hypertrophic Cardiomyopathy in a Brazilian Cohort: Comparison with World Literature

**DOI:** 10.36660/abc.20240399i

**Authors:** Diego Araújo Silva, Edmundo Arteaga-Fernandez, Viviane Tiemi Hotta, Charles Mady, Barbara Ianni, Felix Ramires, Luciano Nastari, Fábio Fernandes, Juliano Novaes Cardoso

**Affiliations:** 1 Brazil Faculdade Santa Marcelina Curso de Medicina, São Paulo, SP – Brazil; 2 Brazil Instituto do Coração do Hospital das Clínicas da Faculdade de Medicina da Universidade de São Paulo, São Paulo, SP – Brazil; 3 Brazil Faculdade de Medicina da Universidade de São Paulo, São Paulo, SP – Brazil; 4 Brazil Hospital Santa Marcelina, São Paulo, SP – Brazil

**Keywords:** Hypertrophic Cardiomyopathy, Risk Factors, Ventricular Tachycardia

## Abstract

**Background:**

In hypertrophic cardiomyopathy (HCM), there is a well-known association between nonsustained ventricular tachycardia (NSVT) and the risk of sudden death.

**Objectives:**

To assess the incidence of NSVT using 24-hour Holter monitoring in patients with HCM in a Brazilian cohort and correlate it with its characteristics and progression.

**Methods:**

This retrospective study of patients with HCM used 24-hour Holter monitoring to assess the presence of long-lasting, fast NSVT (≥ 10 beats and heart rate ≥ 130 bpm) or the presence of at least 3 episodes of NSVT with ≥ 3 beats and heart rate ≥ 120 bpm. Continuous variables were shown as arithmetic means and standard deviations, and categorical variables were shown as absolute and relative frequencies. P < 0.05 was considered significant.

**Results:**

We included 763 patients, 53.5% of whom were male. Their mean age was 52.6 years ± 16.7. NSVT was found in 10% (76 patients). Only 11 (1.4%) of them had NSVT with ≥ 10 beats and heart rate ≥ 130 bpm. There was no difference in the relationship between NSVT and sex, septum > 30 mm, age ≥ 40 years, betablocker dose, and presence of atrial fibrillation. In the group with NSVT, 15-year all-cause mortality was observed in 26.3%, compared to 15.9% in the group without NSVT (p = 0.021).

**Conclusions:**

The presence of NSVT on 24-hour Holter monitoring occurred in 10% of patients. Long-lasting, fast NSVT was rare. The presence of NSVT was associated with higher overall mortality during follow-up.

## Introduction

Hypertrophic cardiomyopathy (HCM) is a disease that presents with left ventricular hypertrophy, with morphological expression restricted to the heart. The presence of HCM involves a wide variety of clinical phenotypes, given that most patients are often asymptomatic or oligosymptomatic. Nonetheless, the disease can cause sudden death, myocardial ischemia, functional limitation, left ventricular systolic dysfunction, atrial fibrillation with increased risk of thrombotic events, and ventricular arrhythmias.^1-8^ It is the most common cause of sudden death in young individuals and athletes, and the majority of episodes are related to ventricular arrhythmia. Risk factors for sudden death include the presence of nonsustained ventricular tachycardia (NSVT), younger age, family history of early sudden death, syncope, septal thickness, left ventricular outflow tract gradient, left atrial size, and quantity of fibrosis. Some studies have associated NSVT with a greater degree of left ventricular involvement and higher mass index, and it is more frequent in cases that have greater wall thickness.^2,5,7-15^

Holter monitoring is recommended for initial assessment and as part of periodic follow-up every 1 or 2 years. This helps assess the risk of sudden cardiac death and guide the management of arrhythmias. Some criteria may make NSVT more relevant, including more frequent, faster, or longer episodes.^4-8^ The incidence of NSVT has been reported in 20% to 54% of patients.^5-10,13-20^

Due to the importance and risks of arrhythmias in HCM, the objective of this study was to assess the incidence of NSVT using 24-hour Holter monitoring in patients with HCM in a Brazilian cohort, in comparison with data from the world literature, and to correlate it with its characteristics and all-cause mortality.

## Methods

This study assessed a retrospective cohort of patients diagnosed with HCM in the outpatient clinic of a tertiary hospital. Echocardiographic diagnosis was defined as diastolic myocardial thickness ≥ 15 mm in any location of the left ventricle, or ≥ 13 mm in individuals with a family history of first-degree relatives with confirmed diagnosis of HCM. The following exclusion criteria were applied: patients under 16 years of age, aortic stenosis, presence of other causes that could justify myocardial hypertrophy, patients who did not have Holter monitoring data from the institution, or patients who did not have complete Holter test available for analysis in the electronic medical records. The sample size was defined by convenience.

Demographic, clinical, and laboratory data were analyzed, in addition to resting electrocardiogram, echocardiogram, and 24-hour Holter. In the echocardiogram, measurements of the cardiac chambers and dimensions were performed in 2-dimensional mode in the longitudinal parasternal view. Clinical follow-up was performed by the attending physicians according to the service routine.

In the 24-hour Holter assessment, we considered long-lasting, fast NSVT episodes (≥ 10 beats with heart rate ≥ 130 bpm) or the presence of ≥ 3 episodes of NSVT, with at least 3 beats and heart rate ≥ 120 bpm. Betablocker use and dosage were evaluated. We considered the use of atenolol ≥ 50 mg/day, propranolol ≥ 40 mg/day, metoprolol ≥ 50 mg/day, or carvedilol ≥ 12.5 mg/day as a high dose.

### Statistical analysis

SPSS V26 (2019), Minitab 21.2 (2022), and Office Excel 2010 were used for statistical data processing. Continuous variables were shown as arithmetic means and standard deviations, and categorical variables were shown as absolute and relative frequencies. We used the unpaired Student’s t test for continuous variables and the chi-square test for categorical variables. The Shapiro-Wilk test was used to test the normality of continuous variables. Survival curves were analyzed with the Kaplan-Meier method, using the log-rank test. The significance level adopted in the statistical analysis was 5%. The study received approval from the institutional ethics committee.

## Results

This study analyzed 2572 patients with myocardial hypertrophy in a database. HCM was confirmed in 1663 patients, and 1593 of them were 16 years of age or older. In this group, 763 had a complete Holter exam available in their medical records that was performed by the institution, thus enabling a more accurate analysis of the data. In the group assessed, 63 patients (8.3%) had a pacemaker, and 52 patients (6.8%) had an implantable cardioverter-defibrillator (ICD). The characteristics of the patients included are described in Table 1.

**Table 1:** – Clinical characteristics (763 patients)

Variable	763 patients % or mean (SD)
Male sex (%)	408 (53.5%)
Age, mean (SD)	52.6 (16.7)
SBP (mmHg), mean (SD)	113.2 (34.7)
DBP (mmHg), mean (SD)	70.5 (21.3)
HR (bpm), mean (SD)	64.8 (20.3)
LVEF, mean (SD)	66% (7.2)
Atrial fibrillation (%)	46 (6.0%)
Glucose, mean (SD)	107.5 (31.1)
Hemoglobin, mean (SD)	14.1 (2.0)
Urea, mean (SD)	41.5 (22.8)
Creatinine, mean (SD)	1.1 (0.6)
Potassium, mean (SD)	4.4 (0.5)
LDL, mean (SD)	108.3 (35.7)
HDL, mean (SD)	47.0 (14.0)
BNP, mean (SD)	464.9 (767.2)

Values are expressed as mean ± SD or frequency and percentage. DBP: diastolic blood pressure; HR: heart rate; LVEF: left ventricular ejection fraction; SBP: systolic blood pressure; SD: standard deviation.

The 24-hour Holter monitoring (Table 2) revealed a mean heart rate of 70.3 ± 11 bpm. Among the patients assessed, 50.5% had mean heart rate < 70 bpm (385 patients), and 13.4% had mean heart rate < 60 bpm (102 patients) on the Holter. The presence of NSVT was found in 76 patients (10%). In only 11 patients (1.4%), Holter revealed NSVT with 10 or more beats and heart rate ≥ 130 bpm. Only 4 patients (0.5%) had NSVT with heart rate ≥ 200 bpm; however, these were short episodes, with a maximum of 4 beats.

**Table 2:** – Results of 24-hour Holter (n = 763)

Parameters	Mean (SD)
Minimum HR (bpm), mean (SD)	49.3 (7.7)
Mean HR (bpm), mean (SD)	70.3 (11.0)
Maximum HR (bpm), mean (SD)	117.9 (24.6)
VES (total), mean (SD)	761.4 (3853.5)
Number of heartbeats in 24 hours, mean (SD)	94,222.4 (16,590.6)
Sustained VT (%)	1 (0.1%)
NSVT (%)	76 (10.0%)

HR: heart rate; NSVT: nonsustained ventricular tachycardia; VES: ventricular extrasystoles; VT: ventricular tachycardia.

Echocardiography revealed that mean left ventricular ejection fraction was 66% ± 7.2%. Left ventricular outflow tract obstruction was found in 219 patients (28.7%), while 43 (5.6%) had septal thickness greater than 30 mm. It was possible to assess the complete prescription in 439 patients, and 351 patients (80%) were using betablockers. Of those who were using betablockers, 212 patients (60.4%) were receiving high doses. The other medications used were verapamil in 42 patients (9.6%), amiodarone in 95 patients (21.6%), and angiotensin-converting enzyme inhibitor or angiotensin receptor blocker in 244 patients (55.6%).

There was no correlation between the presence of NSVT and sex, septal size > 30 mm, age ≥ 40 years, high or low betablocker dose, or presence of atrial fibrillation (Table 3). The Central Illustration displays a summary of the incidence of NSVT in our cohort and its relation with qualitative factors. In the group with NSVT, 15-year all-cause mortality was 26.3% (20 patients), compared to 15.9% (109 patients) in the group without NSVT (p = 0.021). Figure 1 displays the survival curve according to the Kaplan-Meier method.

**Table 3:** – Relation between NSVT and qualitative factors

		With NSVT		Without NSVT		Total		p value
		N	%	N	%	N	%	
Septum greater than 30 mm	No	69	90.8%	651	94.8%	720	94.4%	0.154
	Yes	7	9.2%	36	5.2%	43	5.6%	
High BB dose	No	25	55.6%	202	51.3%	227	51.7%	0.586
	Yes	20	44.4%	192	48.7%	212	48.3%	
AF	No	70	92.1%	647	94.2%	717	94.0%	0.471
	Yes	6	7.9%	40	5.8%	46	6.0%	
Age	< 40 years	15	19.7%	173	25.2%	188	24.6%	0.296
	≥ 40 years	61	80.3%	514	74.8%	575	75.4%	
Death	No	56	73.7%	578	84.1%	634	83.1%	0.021
	Yes	20	26.3%	109	15.9%	129	16.9%	
Sex	Female	39	51.3%	316	46.0%	355	46.5%	0.378
	Male	37	48.7%	371	54.0%	408	53.5%	

AF: atrial fibrillation; BB: betablocker; NSVT: nonsustained ventricular tachycardia.

**Figure 1:** – Survival curve for NSVT. IQD: interquartile deviation; NSVT: nonsustained ventricular tachycardia.

Assessment of cardiac rhythm on resting electrocardiogram and 24-hour Holter monitoring revealed atrial fibrillation rhythm in 46 patients (6.0%). Patients with left atrium size > 40 mm had a higher prevalence of atrial fibrillation (64.8% of patients in sinus rhythm versus 91.1% of patients in atrial fibrillation with left atrium size > 40 mm, p < 0.001).

## Discussion

This study revealed the presence of NSVT in 10% of cases (76 patients), and 15-year overall mortality was higher in the group with NSVT (26.3% versus 15.9%, p = 0.021). There was no statistically significant difference in the relationship between NSVT and sex, septal size > 30 mm, age ≥ 40 years, high or low betablocker dose, or presence of atrial fibrillation. The incidence of long-lasting, fast NSVT was very low, being found in only 1.4% of patients (11 patients), as shown in the Central Illustration.

In the assessment of patients with HCM, the presence of ventricular tachycardia has been associated with sudden death. Holter monitoring is the recommended test to assess the presence of arrhythmias and assist in patient stratification. The presence of sustained ventricular tachycardia is already an indication for ICD implantation.^1,2,7^ Our study revealed that the incidence of sustained ventricular tachycardia observed on 24-hour Holter monitoring was very rare, identified in only 1 patient. It is likely that, because the test lasted only 24 hours, we were not always able to identify this type of arrhythmia. The presence of NSVT is a slightly more frequent finding, according to previously published data.^6-10^

NSVT is one of the major risk factors for sudden cardiac death, in addition to family history of sudden death, unexplained syncope, septal thickness greater than 30 mm, left ventricular ejection fraction < 50%, apical aneurysm, and fibrosis above 15%. Studies have demonstrated that the incidence of NSVT can vary. It has been reported in 20% to 54% of patients with HCM. Our study revealed a lower incidence of this arrhythmia, with 10% presenting NSVT. One of the hypotheses for this difference lies in the characterization of the type of significant NSVT. We should give greater weight to longer, more frequent, and faster arrhythmias, and these criteria were applied in our study. However, some studies include less significant episodes of NSVT.^6-10,13,20^

In a study about NSVT in HCM, Monserrat et al.^6^ assessed 531 patients, and 24-hour Holter monitoring revealed 19.6% with NSVT. NSVT was associated with an increase in sudden death in patients younger than 30 years. The odds ratio for sudden death in patients ≤ 30 years of age with NSVT was 4.35 (95% confidence interval: 1.54 to 12.28; p = 0.006), compared with 2.16 (95% confidence interval: 0.82 to 5.69; p = 0.1) in patients older than 30 years. In our study, the incidence of NSVT was much lower; however, this is related to the characterization of NSVT. We used only more significant NSVT episodes, while the study by Monserrat et al.^6^ considered any type of NSVT. This difference in the criteria used to define significant NSVT may be one of the reasons for the difference in incidence that we found.

Wang et al.^9^ assessed 160 patients who had an ICD implanted, and 94 of them underwent Holter monitoring 24 to 48 hours before implantation. The results of this study revealed that 54% of the total (86 patients) had NSVT, either on the pre-implantation Holter assessment or on ICD monitoring after implantation. One fact that should be taken into consideration is that, in the study by Wang et al.,^9^ the monitoring time was longer, including data from monitoring with an invasive device. Moreover, they selected only patients at higher risk for arrhythmia. Our study included a general population with HCM, and we used 24-hour Holter monitoring, which may justify the difference in the incidence of NSVT. This is another reason that explains the lower incidence of NSVT found in our study. Assessing the incidence of NSVT in patients at high risk for arrhythmia who already require an ICD greatly increases the chances of finding this arrhythmia. Furthermore, performing continuous monitoring with an implantable device will also increase the chances of detecting arrhythmias, as the monitoring time is much longer compared to a 24-hour Holter.

In the European guidelines,^7^ NSVT is calculated in the risk score for sudden death when it presents ≥ 3 consecutive ventricular beats and ≥ 120 bpm with duration < 30 seconds. In the Brazilian device guideline,^5^ NSVT is scored when there are 3 or more episodes, with at least 3 repetitive ventricular beats or at least 1 episode with ≥ 10 beats and heart rate ≥ 130 bpm. In the United States guideline,^2^ NSVT is included as an independent criterion, but with recommendation grade IIb, and the guideline suggests giving greater weight to more frequent (≥ 3), longer (≥ 10 beats), or faster (≥ 200 bpm) NSVT episodes. The Brazilian guideline^1^ suggests emphasizing frequent (≥ 3), longer (≥ 10 beats), and faster (≥ 200 beats per minute) NSVT episodes. Therefore, it is important to identify the presence of more significant NSVT episodes and correlate it with risk. In our study, only 11 patients (1.4%) presented NSVT with 10 or more beats and heart rate ≥ 130 bpm. When we assessed tachycardias with very high heart rate, we found only 4 patients (0.52%) with heart rate ≥ 200 bpm, however, with a maximum of 4 beats. In the United States guideline,^4^ one of the risk criteria for sudden death is the presence of NSVT with heart rate ≥ 200 bpm. As observed in our cohort, the incidence of such fast NSVT episodes was low. Therefore, it is a criterion that we very rarely observe in our daily clinical practice.

Episodes of sustained ventricular tachycardia are more clearly associated with sudden death; however, the data are less robust in demonstrating that the isolated presence of NSVT is an independent risk factor. On the other hand, the risk increases in the presence of risk modifiers, especially left ventricular fibrosis.^8-10,21^ In our study, the presence of NSVT was a marker of worse prognosis in the assessment of overall mortality. Nonetheless, we did not assess the presence of fibrosis, and we did not specifically assess cardiovascular mortality. In a study conducted by Monserrat et al.,^6^ in addition to cardiovascular mortality, they also assessed all-cause mortality. This study revealed that, in patients younger than 30 years, survival was lower in the NSVT group (5-year cumulative survival 61.3% [95% confidence interval: 41.1 to 81.5] with NSVT versus 93.5% [95% confidence interval: 89.4 to 97.6] without NSVT; p = 0.0001). They concluded that NSVT was associated with a substantial increase in all-cause mortality and in the risk of sudden death in young patients with HCM. This risk was independent of the frequency, duration, and heart rate of NSVT episodes. Our study included only overall mortality, showed no difference in relation to age, and revealed higher overall mortality in patients with NSVT. These findings help to establish that the detection of significant NSVT episodes in patients with HCM assists in the risk stratification of severe arrhythmic events, although the decision to implant an ICD should take multiple factors into account.

Betablockers are considered first-line therapy for controlling symptoms, such as dyspnea and chest pain. Non-dihydropyridine calcium channel blockers, such as verapamil and diltiazem, are second-line alternatives for patients who are intolerant or have contraindications to betablockers.^1,2,7^ However, there are no randomized controlled data to support the use of antiarrhythmic drugs for the prevention of sudden death in HCM. In our study, betablocker use was frequent; 80% of patients were using these medications, and 60.4% of patients were receiving high doses. A calcium channel blocker (verapamil) was used in 9.6% of patients (42 patients). In our study, the betablocker dose was not associated with the presence of NSVT.

Left ventricular ejection fraction in HCM is usually normal or increased, and septal thickness greater than 30 mm may be related to disease severity.^2,5,11,21-24^ Our population had a mean left ventricular ejection fraction of 66% ± 7.2%. Moreover, only 5.6% of patients had septal thickness greater than 30 mm, and these patients did not have a greater number of NSVT episodes.

Our study revealed a lower incidence of NSVT when compared to data in the literature.^5-11,13,20-23,25^ This may be related to the type of population studied and to the inclusion criteria for considering NSVT significant. Further prospective studies should be conducted to correlate cardiovascular progression and the presence of complex ventricular arrhythmias in conjunction with other risk factors.

### Limitations

This was a single-center, retrospective study conducted at a tertiary hospital. As it was a convenience sample with no sample size estimate, any statistical inference is exploratory. Given that this was a retrospective study, we were limited by the information assessed. We did not assess comorbidities. In many patients, we did not have access to the complete prescription; therefore, medication use was only assessed in a portion of the patients included. Another limitation is that we did not have access to the cause of death for each patient; therefore, we assessed all-cause mortality.

## Conclusion

This study revealed the presence of NSVT in 10% of cases, and 15-year overall mortality over was higher in the group with this arrhythmia. There was no statistically significant difference in the relationship between NSVT and sex, septal size > 30 mm, age ≥ 40 years, betablocker dose, or presence of atrial fibrillation. The incidence of long-lasting, fast NSVT episodes was very low (1.4% of cases).
